# Prognostic significance and immune characteristics of APOE in gastric cancer

**DOI:** 10.18632/aging.205265

**Published:** 2023-12-04

**Authors:** Xiulan Peng, Zhen Cai, Duansi Chen, Fei Ye, Lifeng Hong

**Affiliations:** 1 Department of Oncology, The Second Affiliated Hospital of Jianghan University, Wuhan, Hubei 430050, China; 2 Department of Operation Room, The Second Affiliated Hospital of Jianghan University, Wuhan, Hubei 430050, China; 3 Department of Oncology, Suizhou Zengdu Hospital, Suizhou, Hubei 441300, China; 4 Department of Radiology, The Second Affiliated Hospital of Jianghan University, Wuhan, Hubei 430050, China; 5 Department of Cardiology, The Second Affiliated Hospital of Jianghan University, Wuhan, Hubei 430050, China

**Keywords:** gastric cancer, APOE, immunotherapy, survival, prognostic, biomarker

## Abstract

Gastric cancer (GC) is a prevalent malignancy affecting the digestive system, and it is the second leading cause of cancer-related mortality worldwide. Immunotherapy presents a potential lifeline for patients with advanced gastric cancer, emphasizing the need to find new molecular targets that improve the response to immunotherapy. In our research, we conducted a comprehensive bioinformatic analysis to investigate the expression profiles of apolipoprotein E (APOE) transcription. Subsequently, we examined the correlation between APOE transcription and the prognosis of GC patients. Additionally, we evaluated the connection between APOE transcription and immune cells abundance. To validate our findings, we conducted immunohistochemistry experiment to ascertain the level of APOE protein in GC patients and assessed its prognostic role in a cohort of 97 GC individuals. Our results revealed that APOE is increased in GC tissues, and APOE displays diagnostic potential in distinguishing GC from normal tissues. Notably, upregulated APOE expression in GC patients is associated with unfavorable overall survival. Differential APOE expression was further observed across different immune subtypes of GC, indicating its involvement in immune cell activation and infiltration. Moreover, we detected increased APOE protein expression in GC tissues, which exhibited a strong correlation with poor survival outcomes. In light of these findings, APOE has become a crucial prognostic molecular with immunomodulatory function in GC. These results underscore the significance of APOE across various cancer types, including GC, and provide valuable insights into its role from both a bioinformatics and clinical perspective.

## INTRODUCTION

Gastric cancer (GC) is a prevalent malignancy affecting the digestive system and ranks as the second leading cause of global cancer-related mortality [1]. *Helicobacter pylori* infection, genetic predisposition, and a diet rich in nitrates and nitrites are common risk factors associated with GC [2]. While various treatment modalities have demonstrated efficacy in managing GC [3], long-term survival outcomes remain unsatisfactory, particularly for individuals with advanced disease. For patients with advanced GC, immunotherapy represents a last resort for prolonged survival [4]. However, only a fraction of patients benefits from this innovative therapeutic approach. Consequently, there is an urgent need to recognize novel molecules that can improve the immunotherapeutic efficacy in GC patients.

APOE, primarily secreted by hepatocytes and macrophages, is essential in lipid metabolism and has been widely researched in cardiovascular disease [5] and Alzheimer's disease [6]. However, emerging evidence indicates its involvement in various human malignancies, including brain tumors [7], bladder cancers [8] and breast cancers [9]. The function of APOE in cancer is multifaceted and depends on the specific situation. For instance, it regulates T-cell suppression in acute myeloid leukemia (AML) [10], while in melanoma, it mediates cytotoxic T-cell responses [11]. Notably, Katsuya et al. [12], discovered that APOE is highly expressed in GC, however, no previous studies have explored the connection between APOE and immune regulation in GC.

We conducted a comprehensive investigation into the transcriptome profiles of APOE across various cancer types. Our focus then shifted to assessing the diagnostic value of APOE specifically in GC. Moreover, we thoroughly scrutinized the interaction between APOE level and the prognosis of GC patients. To substantiate our findings, we utilized sequencing data from TCGA datasets and also validated the survival significance of APOE from GEO database. Additionally, we explored the linkage between APOE level and the abundances of immune cells. Finally, we applied IHC experiments to analyze the expression of APOE in GC and its relationship with the prognosis of GC patients.

## MATERIALS AND METHODS

### Oncomine

Oncomine is a valuable gene chip-based database that facilitates data mining of transcriptional gene expression in various cancer types (http://www.oncomine.org). We utilized Oncomine to analyze APOE mRNA levels in human cancers.

### GEPIA 2

Additionally, we employed GEPIA 2 (http://gepia2.cancer-pku.cn/#index), a website tool that allows for thorough transcriptome analysis using data from TCGA [13]. GEPIA 2 was instrumental in investigating the expression of APOE in GC.

### Kaplan–Meier plotter

To evaluate the prognostic value of APOE in GC, we utilized KM plotter (http://kmplot.com), a website tool containing transcriptome and prognosis data [14]. In our analysis, we categorized GC patients into high and low subgroups according to cut off values as median expression levels of APOE. We then evaluated overall survival (OS) and disease-free survival (DFS) using hazard ratios (HRs) with corresponding 95% confidence intervals (CIs) and log-rank *p*-values.

### TIMER

We utilized TIMER, a website tool with extensive capabilities for analyzing immune cell infiltration levels (https://cistrome.shinyapps.io/timer/) [15]. Initially, we employed the “Diff Exp” module to evaluate expression patterns of APOE across diverse human tumors within TCGA. Subsequently, we utilized the “Gene” module to evaluate the linkage between APOE level and immune cells abundance. Finally, we applied the “Correlation” module to assess the linkage between APOE transcriptome and various gene markers associated with distinct immune cell populations.

### ImmuneCellAI

We employed the Immune Cells Abundance Identifier (ImmuCellAI) (http://bioinfo.life.hust.edu.cn/ImmuCellAI#!/analysis), a cutting-edge algorithm specifically designed for evaluating immune cells concentration, with a particular emphasis on subsets of T cells implicated in tumor initiation and progression [16]. Using the chip sequence data of TCGA-STAD, we leveraged ImmuCellAI to examine the relative concentration of 24 types of immune cells between the low and high APOE expression groups.

### Immune cells and response to immunotherapy

To assess the enrichment scores for GC individuals, we employed the single-sample gene set enrichment analysis (ssGSEA) approach. Specifically, we utilized the R software [17] and R package ‘GSVA’ to perform the ssGSEA analysis [18]. Based on the median expression value of APOE, we categorized the GC individuals into low and high APOE group in the TCGA-STAD. Subsequently, we calculated the normalized enrichment scores for 28 different immune cell types, as previously described [19]. Furthermore, we evaluated the activities of 7 cancer-related steps [20] and oncogenic pathways [21] using ssGSEA. We employed Spearman correlation analysis to examine the correlations between APOE level and these gene sets in GC patients.

### Collection of GC tissues

In this study, a comprehensive collection of 180-spot tissue array chips was obtained from Shanghai Outdo Biotech, Ltd. The tissue array chip contains a total of 97 GC samples and 83 corresponding normal tissues. Importantly, these samples were accompanied by 5 years of prognosis data. All patients’ samples were collected with their permission, and our research protocol received ethical approval from the Ethics Committee of The Second Affiliated Hospital of Jianghan University.

### Immunohistochemical assay

Immunohistochemistry (IHC) staining was conducted as previously reported [22]. In brief, tissue slices were first deparaffinized and gradually rehydrated using a series of graded ethanol solutions. To facilitate antigen retrieval, the deparaffinized tissue slices were subjected to boiling in 10 mM citrate buffer (pH 6.0) for 20 minutes. Afterwards, the tissue slices were incubated with 3% H_2_O_2_ solution to quench the activity of endogenous peroxidase. The slices were immersed with the specific primary antibody (1:500 dilution, ab109117, Abcam). Visualization of the antibody-antigen interaction was achieved through DAB, followed by counterstaining with hematoxylin.

### Immunohistochemical scoring

In our research, a semi-quantitative scale based on staining intensity and staining extent was employed by two experienced researchers (Xiulan Peng and Duansi Chen) independently. The staining density was categorized into four groups: 0 point (negative), 1 point (weakly stained), 2 points (moderately stained), and 3 points (strongly stained). Staining extent was assessed and assigned scores as follows: 1 point (stained area 1–25%), 2 points (stained area 26–50%), 3 points (stained area 51–75%), and 4 points (stained area 76–100%). The staining extent and intensity score were multiplied to calculate the IHC score of each tissue sample. We categorized GC patients into low and high APOE protein groups on the basis of the median value of APOE IHC scores among all these GC patients.

### Statistical analysis

We completed the data analysis using SPSS software (version 21) and R software (version 3.5.1). Spearman’s correlation analysis was employed to examine the linear connection between APOE transcriptome and immune cell infiltration. ROC analysis was executed to determine the diagnostic power of APOE mRNA for the discrimination of GC tissues and normal gastric tissues, which was measured by area under the curve (AUC) with 95% CIs. The results of ROC analyses were displayed in the ROC curves. The relationship between prognosis and APOE level was shown by Kaplan-Meier survival analysis, and the difference between two groups was examined by log-rank tests. *P*-value < 0.05 is statistically significant.

### Data availability statement

The datasets used and analyzed during the current study are available from the corresponding author on reasonable request.

## RESULTS

### APOE is highly expressed in several cancerous tissues

**Figure 1:**
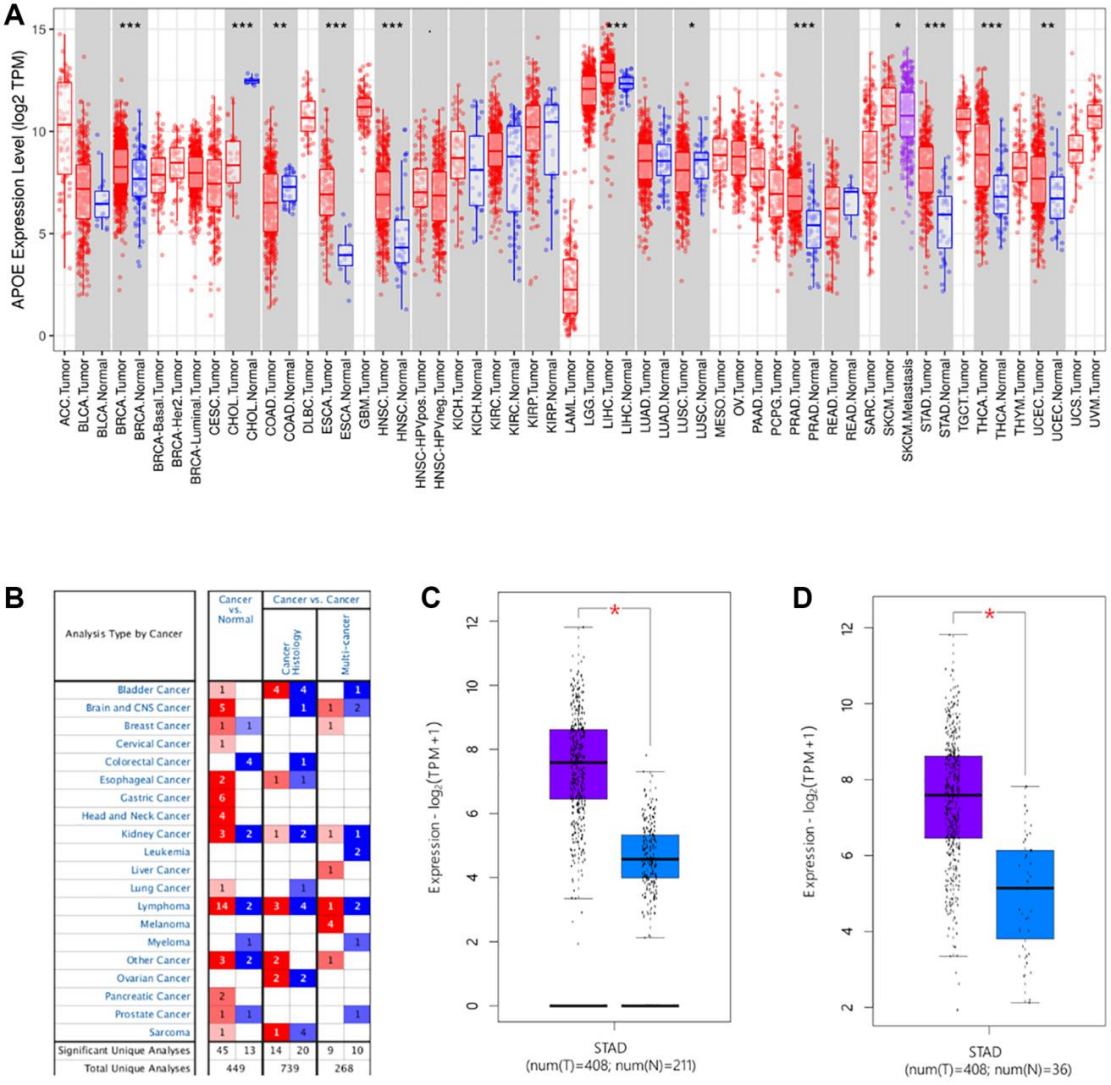
**The transcription levels of APOE in human cancers.** APOE mRNA expression in pan-cancer (**A**, **B**) and gastric cancer (**C**, **D**).

To examine APOE transcriptome level in diverse human cancers, we conducted an analysis using TIMER (Figure 1A) and Oncomine (Figure 1B). Our findings revealed that APOE exhibited significantly higher expression in several cancer tissues, including stomach adenocarcinoma (STAD). Subsequently, we quantitatively examined the transcription levels of APOE in GC, based on sequencing data obtained from the TCGA database. Consistently, we observed up-regulation of APOE mRNA in GC tissues (Figure 1C, 1D).

**Figure 2:**
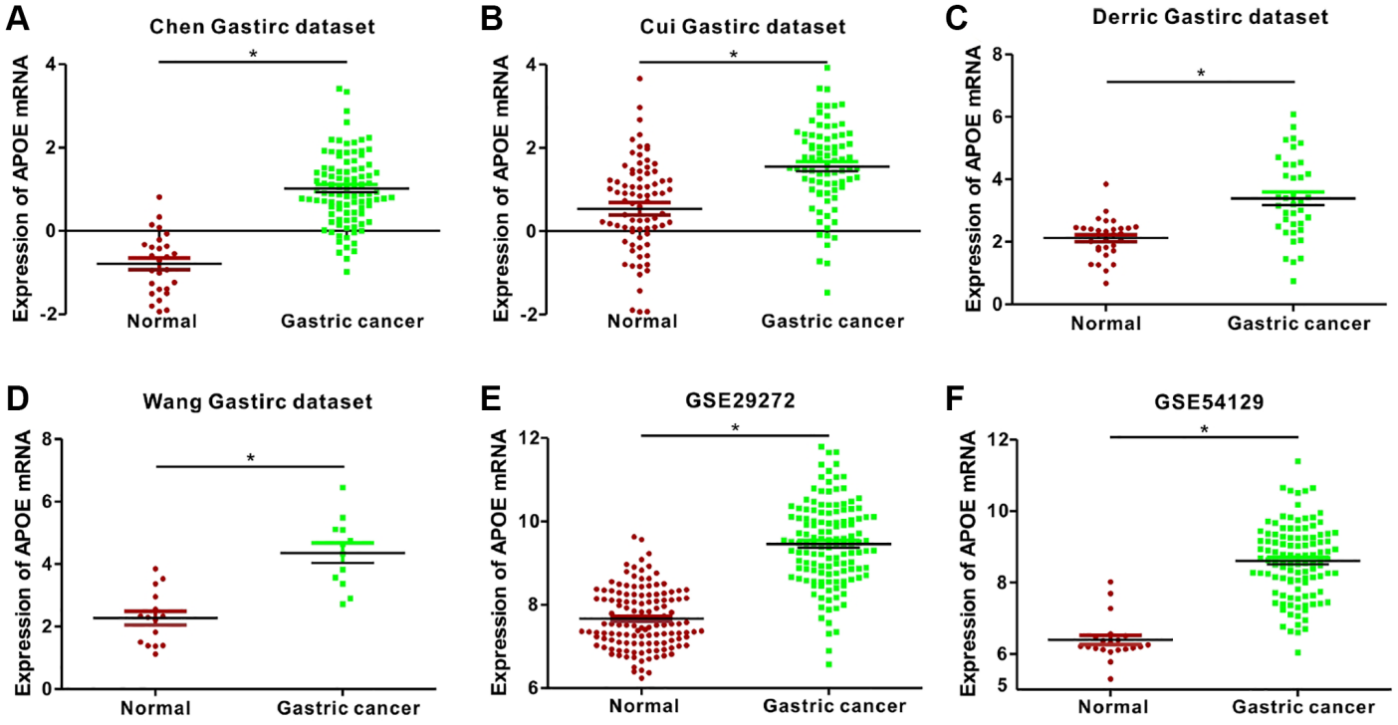
**Analysis of APOE mRNA expression in normal and gastric cancer (GC) tissues from 2 public databases.** APOE mRNA levels are significantly lower (*P* < 0.0001) in normal gastric mucosal than that in gastric cancer tissue samples in the (**A**) Chen (Normal = 29; Tumor = 83), (**B**) Cui (Normal = 80; Tumor = 80), (**C**) Derric (Normal = 31; Tumor = 38), and (**D**) Wang (Normal = 15; Tumor = 12) Gastric datasets from the Oncomine database; and (**E**) GSE29272 (Normal = 134; Tumor = 134) and (**F**) GSE54129 (Normal = 21; Tumor = 111) datasets from the GEO databases.

**Figure 3:**
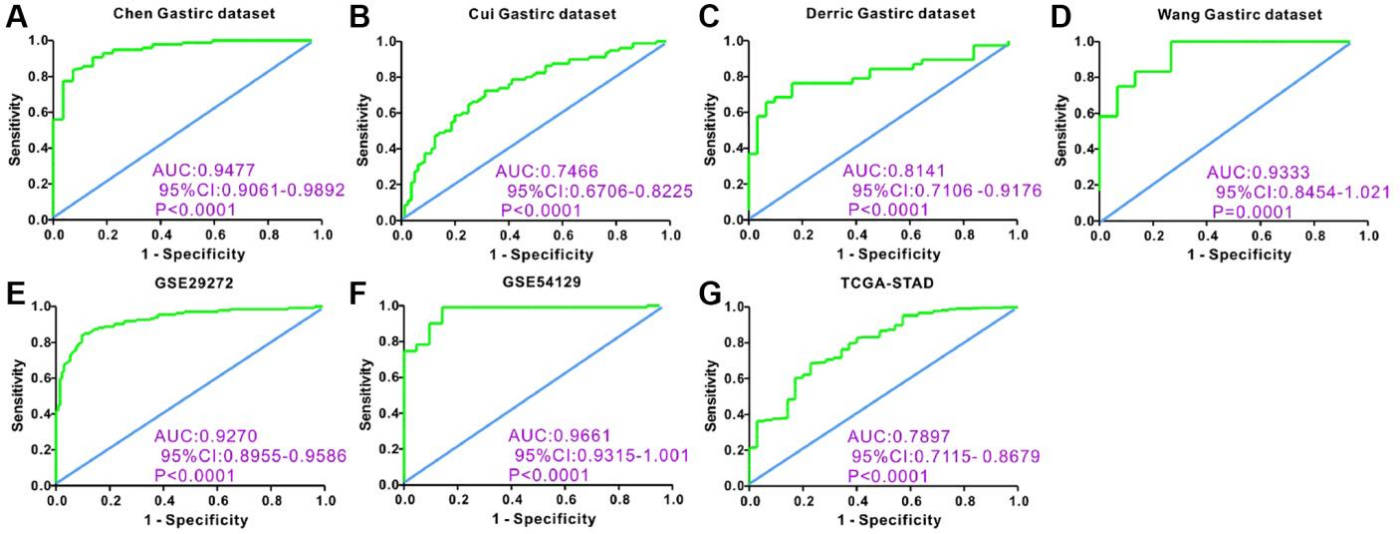
**Receiver operating characteristic (ROC) curve analysis to determine diagnostic relevance of APOE mRNA levels in GC patients.** ROC curve analysis of APOE mRNA levels in the (**A**) Chen (AUC = 0.9477), (**B**) Cui (AUC = 0.7466), (**C**) Derric (AUC = 0.8141) and (**D**) Wang (AUC = 0.9333) Gastric datasets from the Oncomine database; (**E**) GSE29272 (AUC = 0.9270) and (**F**) GSE54129 (AUC = 0.9661) datasets from the GEO databases; and (**G**) STAD dataset (AUC = 0.7897) from the TCGA database; The receiver operating characteristic (ROC) curve analysis to determine diagnostic relevance of APOE mRNA levels in GC patients.

Additionally, we analyzed APOE expression pattern in GC, utilizing sequencing data from the GEO datasets. Remarkably, the results remained consistent with those obtained from the TCGA datasets (Figure 2). Moreover, we protracted ROC curve to explore the diagnostic value of APOE transcription levels in GC, according to data derived from both GEO and TCGA databases (Figure 3). Notably, the highest diagnostic value (AUC = 0.9661, 95% CI: 0.9315–1.001) for APOE mRNA expression was observed in the GSE54129 dataset (Table 1).

**Table 1:** Diagnostic performance of APOE mRNA for gastric cancer.

**Dataset**	**AUC**	**Cut-off value**	**Sensitivity**	**Specificity**	***P*-value**
Chen gastric	0.9477	−0.0475	90.82%	85.19%	<0.0001
Cui gastric	0.7466	1.134	72.50%	68.75%	<0.0001
Derric gastric	0.8141	2.437	76.32%	80.65%	<0.0001
Wang gastric	0.9333	3.374	83.33%	86.67%	0.0001
GSE29272	0.927	8.389	88.06%	84.33%	<0.0001
GSE54129	0.9661	7.291	90.09%	90.48%	<0.0001
TCGA	0.7897	10.67	76.39%	65.71%	<0.0001

### APOE transcriptome level is related to GC patients’ prognosis

**Figure 4:**
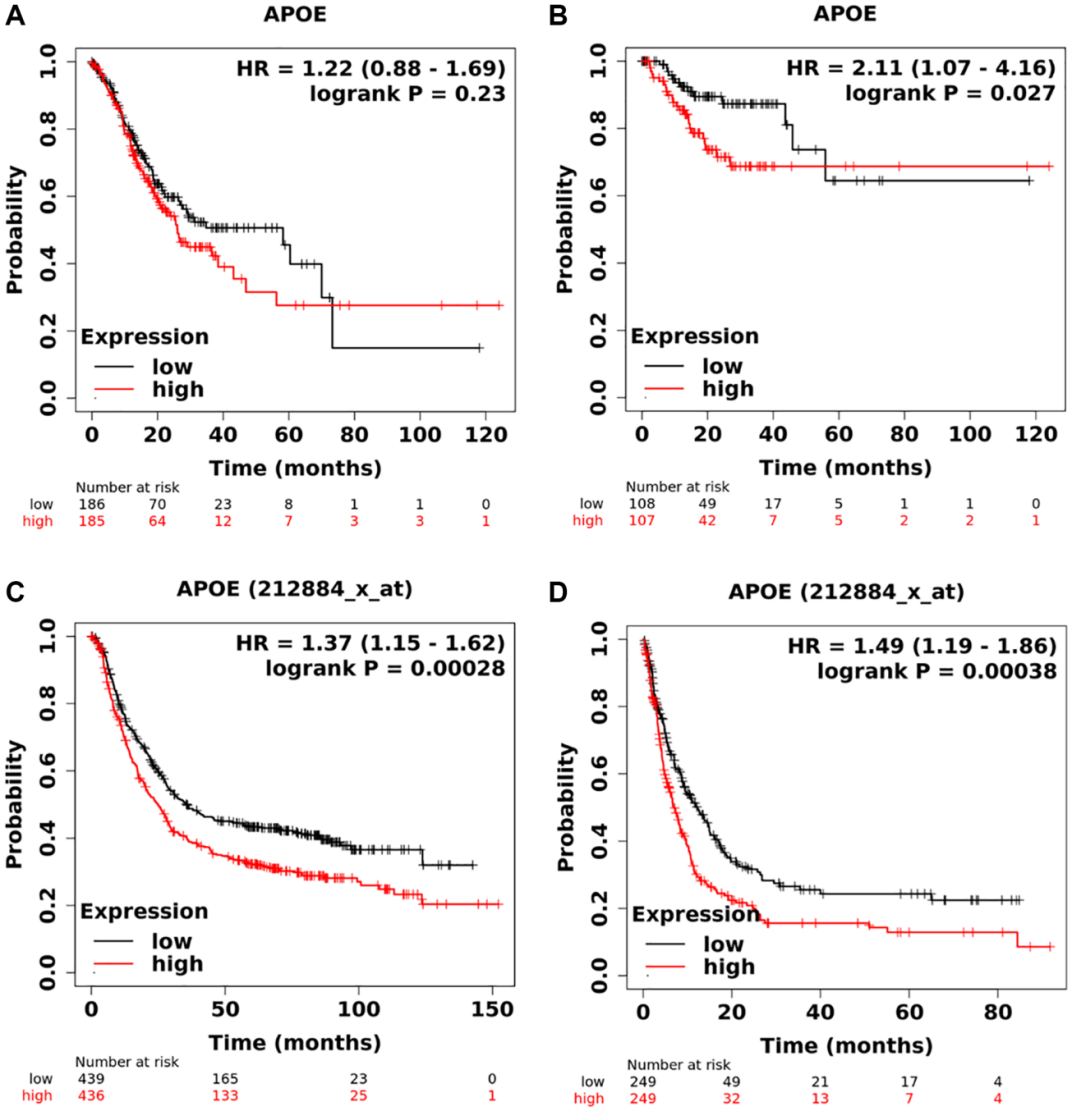
**Survival analysis of APOE mRNA in gastric cancer.** Low levels of APOE are correlated with longer overall time (**A**) and disease-free survival time (**B**) based on TCGA dataset. Low levels of APOE are related to longer overall time (**C**) and disease-free survival time (**D**) based on GEO database.

Kaplan-Meier plotter database was utilized to investigate the prognostic value of APOE in GC. Initially, we examined the TCGA-STAD and observed that GC patients with lower expression of APOE displayed a tendency towards better OS (HR = 1.22, 95% CI: 0.88–1.69, *P* = 0.23, Figure 4A) and DFS (HR = 2.11, 95% CI: 1.07–4.16, *P* = 0.027, Figure 4B). Consistently, in the GEO cohort we also found that lower expression of APOE was linked to improved OS (HR = 1.37, 95% CI: 1.15–1.62, *P* = 0.00028, Figure 4C) and DFS (HR = 1.49, 95% CI: 1.19–1.86, *P* = 0.00038, Figure 4D). Additionally, we investigated the correlation between APOE expression and prognosis with diverse clinical parameters (Table 2).

**Table 2:** Subgroup survival analysis of APOE mRNA in patients with gastric cancer.

**Clinical factors**		**Overall survival**			**Progression-free survival**		
		**HR**	**95% CI**	***P*-value**	**HR**	**95% CI**	***P*-value**
Gender	Female	1.83	1.28–2.6	0.00074	2.21	1.43–3.41	0.00025
	Male	1.3	1.05–1.6	0.017	1.3	1.01–1.68	0.047
Treatment	Surgery	1.37	1.03–1.83	0.03	1.66	1.21–2.27	0.0014
	5-Fu based chemotherapy	0.96	0.68–1.36	0.83	1.26	0.89–1.79	0.2
	other	1.26	0.52–3.04	0.61	1.29	0.53–3.15	0.57
HER2	Negative	1.4	1.1–1.75	0.0036	1.39	1.04–1.85	0.023
	Positive	1.29	1.0–1.68	0.051	1.74	1.22–2.48	0.0019
T stage	T1 stage	–	–	–	–	–	–
	T2 stage	1.34	0.87–2.05	0.18	1.54	0.98–2.41	0.057
	T3 stage	1.26	0.9–1.78	0.19	1.45	0.98–2.13	0.059
	T4 stage	1.71	0.73–4.01	0.21	1.4	0.54–3.64	0.49
N Stage	N0 stage	0.85	0.37–1.93	0.69	0.8	0.25–2.52	0.7
	N1-3 stage	1.79	1.37–2.34	1.3 × 10^−5^	1.9	1.42–2.53	9.3 × 10^−6^
M stage	M0 stage	1.36	1.03–1.08	0.029	1.53	1.14–2.07	0.0049
	M1 stage	1.12	1.63–1.98	0.71	1.94	0.92–4.1	0.079
Lauran classification	Intestinal	1.94	1.41–2.68	3.6 × 10^−5^	1.83	1.2–2.77	0.0041
	Diffuse	1.55	1.1–2.19	0.011	1.59	1.08–2.33	0.018
	Mixed	0.76	0.27–2.09	0.59			
Differentiation	Poor	0.98	0.66–1.46	0.92	0.92	0.49–1.72	0.79
	Moderate	1.87	0.97–3.6	0.057	4.63	1.57–13.69	0.0027
	Well	2.31	0.97–5.51	0.053	–	–	–
TNM stage	Stage I	0.68	0.25–1.88	0.46	0.52	0.1–2.71	0.43
	Stage II	1.45	0.79–2.65	0.23	2.07	1.05–4.11	0.033
	Stage III	1.49	1.12–1.99	0.006	1.68	1.08–2.54	0.019
	Stage IV	1.08	0.74–1.59	0.68	1.22	0.78–1.91	0.38

### APOE is connected with immune activation and infiltration in GC

**Figure 5:**
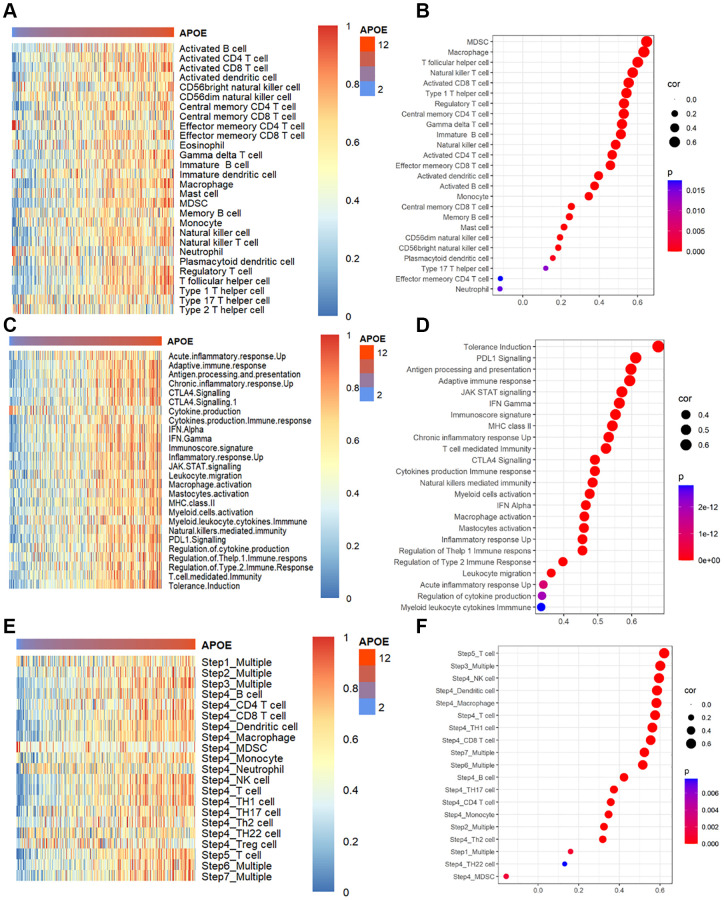
**APOE is associated with immune infiltration and immune activation in GC.** (**A**) Heatmap displayed APOE mRNA level associated relative abundance of 28 immune cells in GC. (**B**) The relationship between the mRNA level of APOE and 28 immune cells in GC. (**C**) Heatmap showing relative association between APOE and 25 immunity-related gene sets. (**D**) The relationship between 25 immunity-related gene sets and APOE in GC. (**E**) Heatmap showing relative association between APOE and steps of the cancer immunity cycle. (**F**) The relationship between steps of the cancer immunity cycle and APOE in GC.

It is well known that the survival of GC is linked to the activation and infiltration of immune cells in the tumor microenvironment (TME). Previous studies have reported APOE enhances the migration of GC cells by being transferred from TAMs to GC cells [23]. Hence, we aimed to investigate the connection between APOE level and immune cells abundance. As depicted in Figure 5A, immune cell scores tended to increase with higher APOE mRNA expression levels. We applied Spearman’s correlation analysis to assess the connection of APOE transcriptome level and immune cells, revealing significant correlations with T cells, B cells, Macrophages, and DCs (Figure 5B). Moreover, we examined the connection between APOE transcriptome level and immune phenotypes, as depicted by a heat map showing a positive trend between immune phenotypes and APOE mRNA expression level (Figure 5C). The correlation analysis further highlighted close associations between APOE mRNA level and tolerance induction (r = 0.68, *P* < 0.0001), PDL1 signaling (r = 0.61, *P* < 0.0001), antigen processing and presentation (r = 0.60, *P* < 0.0001), adaptive immune response (r = 0.59, *P* < 0.0001), and JAK STAT signaling (r = 0.57, *P* < 0.0001) (Figure 5D). Moreover, we evaluated the impact of APOE mRNA expression on the cancer immunity cycle, which encompasses seven critical steps representing the anticancer immune response. In subgroups with higher APOE mRNA expression, most steps of the cancer immune cycle are activated (Figure 5E, 5F).

### APOE is associated with immune activation and immune infiltration in GC

**Figure 6:**
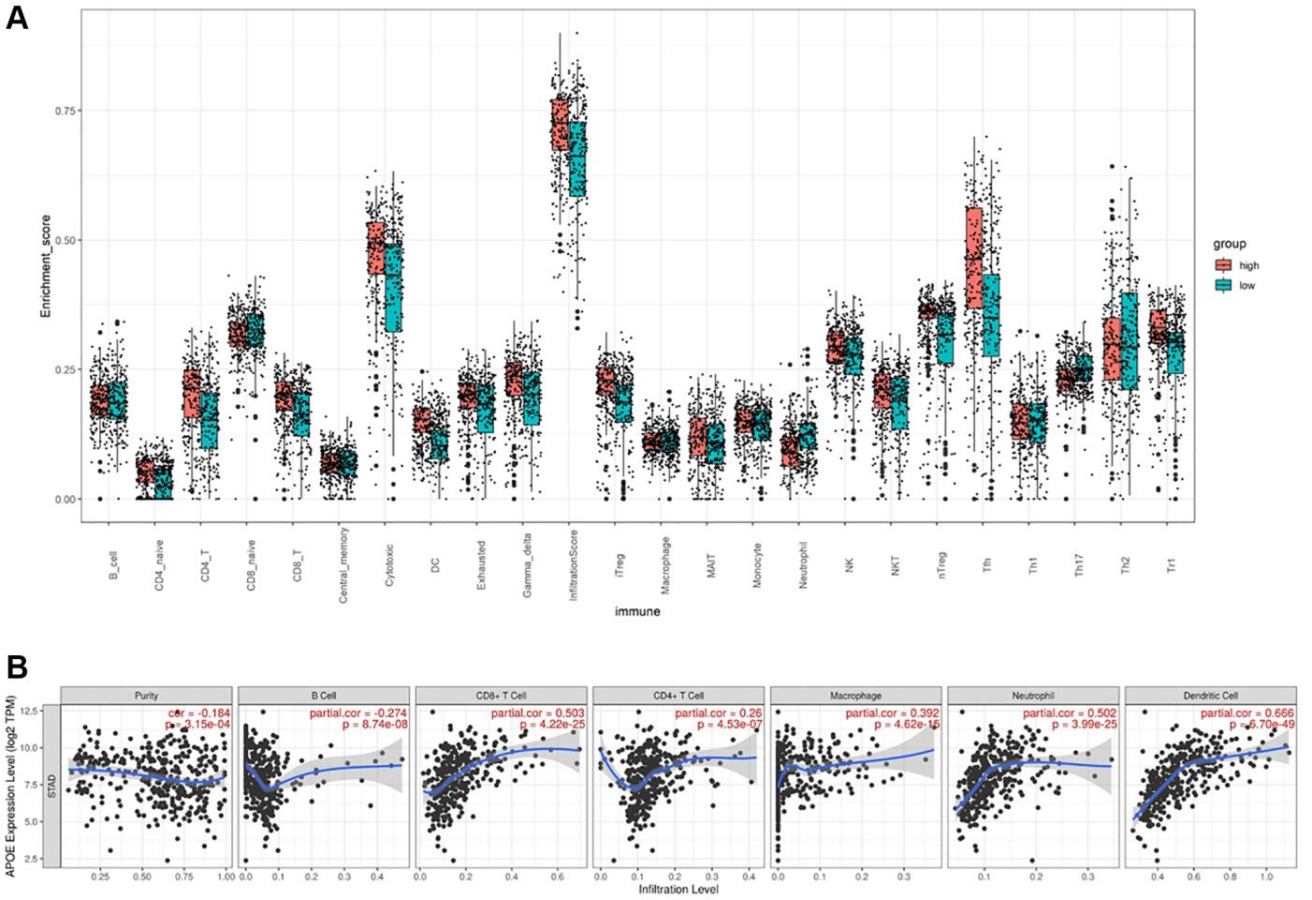
**APOE is associated with immune cell infiltration in GC.** (**A**) The comparison of TILCs in APOE low and high subgroups. (**B**) The association between APOE and immune cells.

We conducted an analysis to examine the relative abundances of 24 types of immune cells in GC using ImmuCellAI to examine the correlation between APOE and immune cells. As depicted in Figure 6A, there were significant differences in the types and concentration of immune cells in patients with low and high APOE subgroups. Specifically, the APOE high subgroup exhibited higher proportions of CD4 naïve cells, CD4 T cells, CD8 T cells, Cytotoxic cells, DC cells and etc. Conversely, the APOE low subgroup demonstrated higher proportions of CD8 naïve cells, Neutrophils, and Th17 cells.

To thoroughly understand the intricate interplay between APOE and the immune response, we conducted an extensive analysis utilizing the TIMER to scrutinize the correlations between APOE transcriptome level and various immune signatures in GC. Specifically, we investigated the correlations between APOE transcriptome level and the abundances of six key immune cell types. Remarkably, our findings unveiled significant positive correlations between APOE transcriptome level and the abundances of critical immune players in GC. Notably, APOE expression displayed a marked positive correlation with the abundance of DCs (r = 0.666, *P* < 0.0001), CD8+ T cells (r = 0.503, *P* < 0.0001), and neutrophils (r = 0.502, *P* < 0.0001) (Figure 6B). These outcomes shed light on the underlying role of APOE in modulating the immune landscape within the context of GC.

**Figure 7:**
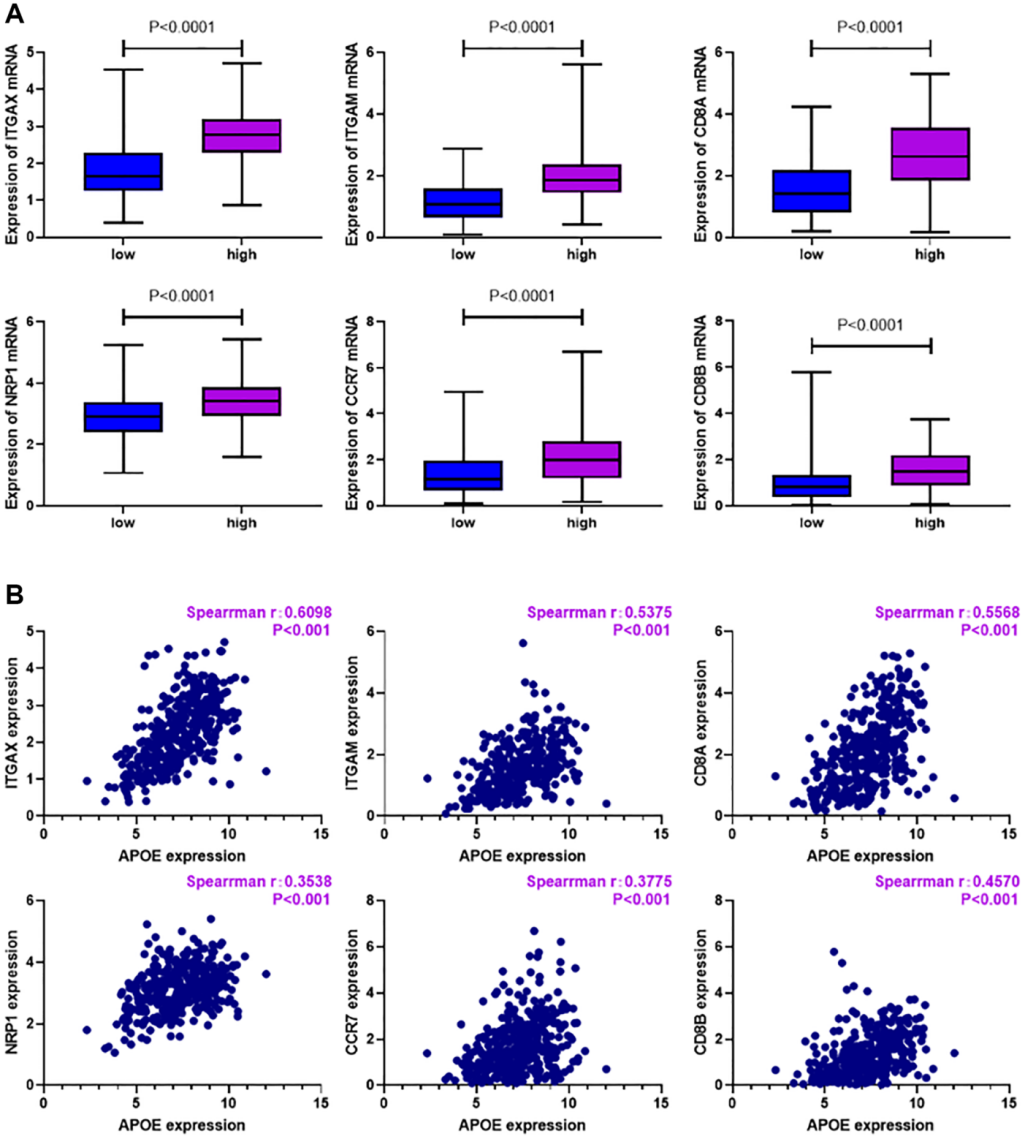
**Relationship between immune cell markers and APOE transcription in GC.** (**A**) Levels of ITGAX (*P* < 0.0001), NRP1 (*P* < 0.0001), ITGAM (*P* < 0.0001), CCR7 (*P* < 0.0001), CD8A (*P* < 0.0001) and CD8B (*P* < 0.0001) were significantly higher in APOE higher group. (**B**) Levels of ITGAX (r = 0.6098, *P* < 0.0001), NRP1 (r = 0.3538, *P* < 0.0001), ITGAM (r = 0.5375, *P* < 0.0001), CCR7 (r = 0.3775, *P* < 0.0001), CD8A (r = 0.5568, *P* < 0.0001) and CD8B (r = 0.457, *P* < 0.0001) are positively correlated with APOE expression in GC.

In addition, we sought to assess the connection between APOE transcriptome level and specific markers of immune cells. Notably, we investigated the expression levels of ITGAX and NRP1 as dendritic cell (DC) markers, ITGAM and CCR7 as neutrophil markers, as well as CD8A and CD8B as markers for CD8+ T cells. Our results, as illustrated in Figure 7A, revealed a substantial increase in the levels of ITGAX, NRP1, ITGAM, CCR7, CD8A, and CD8B within the APOE high group in comparison to the APOE low group (*P* < 0.0001). This observation emphasizes the potential association between APOE and these immune cell markers in GC. Furthermore, through correlation analysis, we demonstrated a positive correlation between the mRNA expression level of APOE and ITGAX, NRP1, ITGAM, CCR7, CD8A, and CD8B in GC (Figure 7B) (*P* < 0.0001). These findings further emphasize the potential role of APOE in influencing the level of these markers of immune cells in GC.

### Clinical validation with 97 GC cases

**Figure 8:**
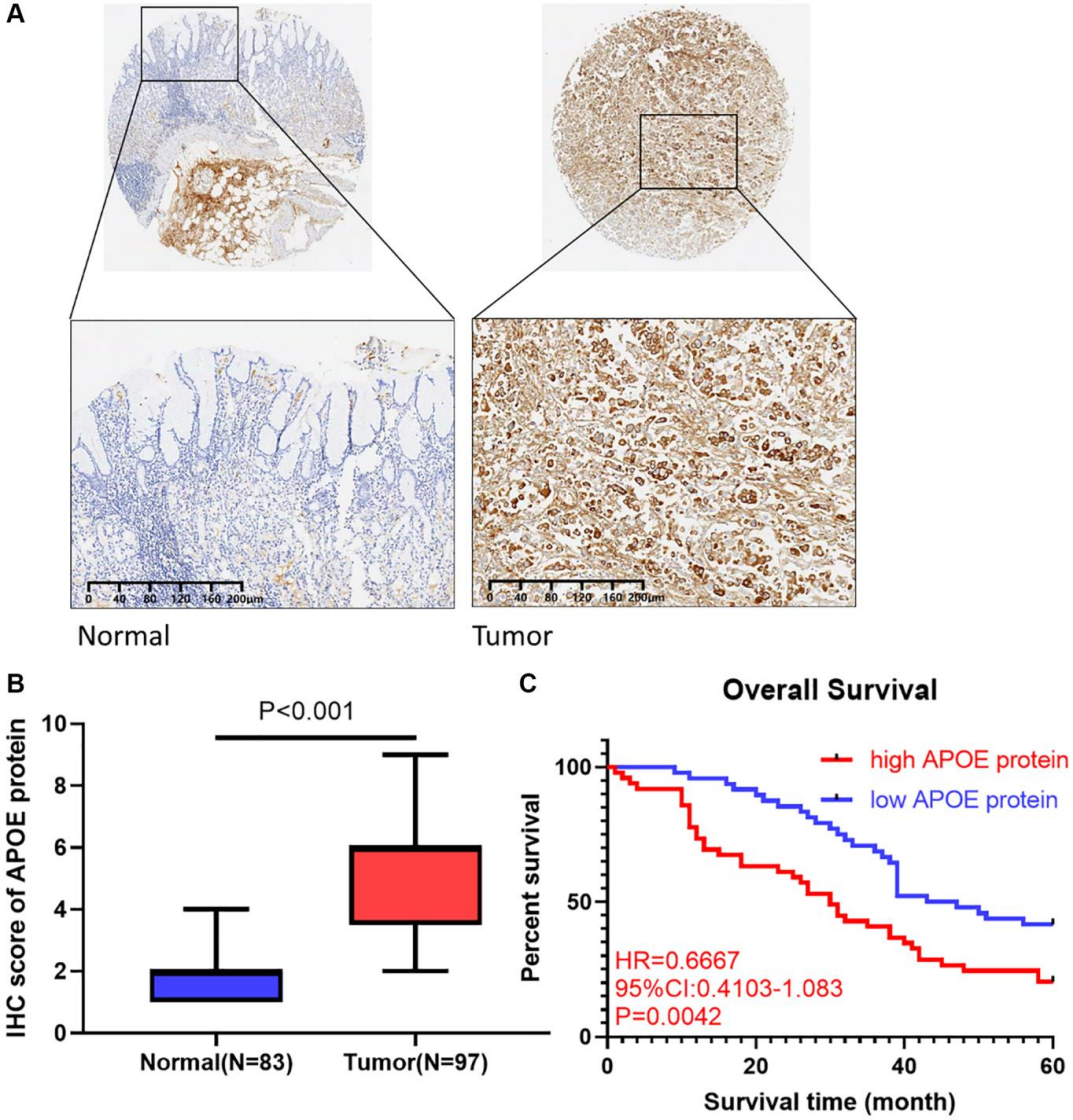
**APOE is upregulated in GC clinical samples and correlated with shorter survival in GC patients.** (**A**) Immunohistochemical staining of normal and gastric cancer tissues with anti-APOE antibody. (**B**) Quantitative analysis of APOE staining shows significantly H-score in gastric tumor samples compared with adjacent normal tissues (83 normal tissues and 97 tumor samples). (**C**) GC patients with APOE over-expression displayed less favorable overall survival than those with low APOE expression.

To ascertain APOE protein expression pattern in GC, we employed a cohort comprising 97 GC tissues obtained from Shanghai Qutdo Biotech Company. Through semiquantitative analysis, we observed a remarkable increase in the intensity of APOE staining in GC tissues (Figure 8A, 8B), indicating a potential role for APOE in the development or progression of GC.

Utilizing survival analysis, we scrutinized the influence of APOE protein expression on GC patients’ OS. Remarkably, our findings disclosed that GC individuals with high protein level of APOE exhibited a prolonged OS time (HR = 0.6667, 95% CI: 0.4103–1.083, *P* = 0.0042, Figure 8C). Our findings highlighted high APOE protein level may serve as a prognostic indicator for more favorable survival outcomes in GC patients. Furthermore, our assessment of APOE protein expression aligns with the earlier analysis of APOE transcription levels, further substantiating that increased APOE level, at both the protein and transcriptional levels, is associated with more favorable survival outcomes in GC patients. Collectively, our findings underscore the underlying significance of APOE as a risk factor and prognostic marker in GC.

## DISCUSSION

In our research, we aimed to recognize new markers that can help predict and improve the response of GC individuals to immunotherapy. Results show that APOE is upregulated in GC and has a diagnostic value in distinguishing GC from normal tissues. What’s more, high level of APOE in GC individuals predicts disappointing overall survival time. Using ssGSEA algorithm, we found that APOE is connected with immune cell activation and infiltration in GC, such as DC, macrophage and CD8^+^ T cell. Finally, we observed elevated levels of APOE protein in GC tissues, which predicted a poor prognosis for GC patients. In conclusion, our study confirms the promising promise of APOE in immunotherapy in GC patients.

APOE has emerged as a crucial player in various cancer types, with diverse roles in each context. In the case of pancreatic ductal adenocarcinoma (PDAC) [24], increased levels of apolipoprotein E (APOE) have been associated with immune suppression, and higher serum APOE levels have been correlated with poorer patient survival. Studies in a PDAC mouse model have further demonstrated that ApoE^−/−^ mice exhibit elevated levels of CD8+ T cells within tumors compared to wild-type mice. In GC [23], a research revealed the mechanism by which exosome-mediated APOE proteins are transferred from tumor-associated macrophages to tumor cells. This process contributes to the migration of GC cells, shedding light on the multiplex interaction between APOE and TME in the context of GC. While in ovarian cancer [25], the expression of APOE in nuclei has been found to be significantly linked to a superior prognosis in patients presenting peritoneal effusion at the time of diagnosis. This highlights a potential prognostic significance of nuclear APOE expression in ovarian cancer patients. Furthermore, in lung cancer [26], APOE has been implicated in promoting cancer proliferation and migration, and its overexpression has been linked to a more aggressive features in patients with lung adenocarcinoma. In hepatocellular cancer (HCC) [27], APOE transcription was linked to relatively lower levels of immune infiltrates and activation in hepatocellular cancer, while APOE hypermethylation displayed a closer association with immune cell presence in this context. Collectively, these findings underscore the multifaceted role of APOE in different cancer types, contributing to our understanding of its involvement in cancer development, progression, and patient outcomes.

The tumor microenvironment (TME) is a multiplex ecosystem comprising diverse immune cells that can either restrain or facilitate tumor progression, making them a double-edged sword [28]. Increasing evidence underscores their value in predicting prognosis and the effectiveness of immunotherapy. Zeng et al. [29] developed an open-source TMEscore R package to quantify the tumor microenvironment (TME) and found it is a promising predictive factor for GC patients, which is consistent with our conclusion: GC is closely related to the immune microenvironment. In our analysis, we disclosed a positive correlation between APOE expression and several immune cell types in GC tissues, including CD8+ T cells, dendritic cells, and neutrophils. Our enrichment analysis further revealed that APOE is predominantly involved in T cell activation, suggesting a pivotal role for APOE in immune response regulation in GC. Remarkably, APOE expression exhibited significant correlations with various immune markers across different immune cell types in GC. These findings provide evidence implying that APOE contributes to the regulation of tumor immune and has potential as a molecular target for GC immunotherapy. However, the precise interplay by which APOE works in the tumor microenvironment needs further study.

Our research sheds light on the relationship between APOE and GC; however, there are two limitations that should be acknowledged in our analysis. Firstly, we focused on exploring the mRNA expression of APOE in GC and validated our bioinformatic findings using a clinical cohort. However, the underlying mechanisms by which APOE influences tumor growth and metastasis remain unexplored in our study. Secondly, the clinical cohort included a limited number of GC patients, and information of immunotherapy was inaccessible. Therefore, future studies are urgently needed to elucidate the mechanism of action of APOE and its predictive value for response to GC immunotherapy.

## CONCLUSION

In summary, our study provides a comprehensive investigation of APOE mRNA and protein expression patterns, its prognostic significance and correlation with immune cells by integrating bioinformatics analysis and a clinical cohort. Our findings support that APOE serves as a dependable prognostic indicator in GC and highlights its potential as a novel target for immunotherapy. Nonetheless, further biological research focused on APOE in GC is needed to confirm our current results and uncover the underlying mechanisms of its involvement in GC development and therapeutic response.
